# Chloroquine Downregulation of Intestinal Autophagy Changed Intestinal Microbial Community Compositions and Metabolite Profiles in Piglets

**DOI:** 10.3390/vetsci11080333

**Published:** 2024-07-25

**Authors:** Xueling Gu, Simeng Liao, Meng Li, Jing Wang, Bie Tan

**Affiliations:** 1 Key Laboratory of Hunan Province for the Products Quality Regulation of Livestock and Poultry, College of Animal Science and Technology, Hunan Agricultural University, Changsha 410128, China; guguxueling@sina.com (X.G.); liaosimeng23@hunau.edu.cn (S.L.); jingwang023@hunau.edu.cn (J.W.); 2 Yuelushan Laboratory, Changsha 410128, China; zllwithann@gmail.com

**Keywords:** chloroquine, autophagy, oxidative stress, gut microbiota, ileal metabolite profiles, weaned piglets

## Abstract

**Simple Summary:**

Weaning stress is a critical factor contributing to diseases and even mortality in piglets during the weaning process. Research indicates that inhibition of intestinal autophagy to a certain extent can alleviate the decline in growth performance associated with weaning stress by enhancing the management of intestinal oxidative stress and inflammation. Interestingly, gut microbes serve an irreplaceable role in the regulation of autophagy. Therefore, we speculate that intestinal microbes may play a direct or indirect role in the process of intestinal autophagy inhibition, alleviating weaning stress in piglets. In this study, we utilized autophagy inhibitors (chloroquine, CQ) or activators (rapamycin, RAPA) to modulate the intestinal autophagy level in weaned piglets. We then investigated how changes in autophagy impacted the composition of intestinal microbes and their metabolites in these piglets, allowing us to explore the effects of autophagy from a microbial perspective. We found that the level of autophagy affected the composition of intestinal microbes and metabolites of weaned piglets, which may be one of the key factors in how autophagy alleviates weaning stress in piglets. Our findings also provide some insights to may guide the future application of autophagy inhibitors in piglets’ diets.

**Abstract:**

Our previous study demonstrated that moderate inhibition of intestinal autophagy was beneficial to alleviate early weaning stress in piglets, but the detailed mechanism behind this was unclear. Microbiota-mediated enterocyte autophagy helps maintain intestinal homeostasis. This study investigated the effects of inhibition or activation of autophagy in intestinal microbial community compositions and metabolite profiles in piglets. Eighteen 24-day-old weaned piglets were divided into three groups (each treatment of six piglets) and treated daily with rapamycin (RAPA), chloroquine (CQ) or a control volume of normal saline (CON group). Before the formal trial, the piglets were allowed to acclimatize for 3 days, and then the trial period was 14 days. Collected samples from the ileum and colon underwent 16S rRNA gene sequencing and metabolite analysis. Significant differences in microbial composition were observed in both the ileum and colon of the RAPA and CQ groups compared to the CON group (*p* < 0.05). In addition, the relative levels of abundance of *Peptostreptococcus*, *Fusobacterium*, *Dialister*, *Selenomonas* and *Oceanobacillus* in the ileum and *Porphyromonas*, *Bacteroides*, *unidentified_Lachnospiraceae*, *Akkermansia*, *Sharpea*, *Peptococcus*, *Pseudoalteromonas*, *Peptoclostridium* and *unidentified_Acidobacteria* in the colon were improved in piglets fed the RAPA diet, whereas the relative levels of abundance of *Turicibacter*, *Rickettsiella* and *Sarcina* in the ileum and *Roseburia* and *Kroppenstedtia* in the colon were enhanced in the CQ group (*p* < 0.05). Meanwhile, metabolomic analysis showed that there were significant differences in metabolites among all groups (*p* < 0.05), and KEGG enrichment analysis revealed that differential metabolites were mainly enriched in the ABC transporters and biosynthesis of amino acids pathways. Furthermore, these metabolites were closely related to differential microorganisms (*p* < 0.05). Overall, autophagy inhibition regulates the composition of intestinal microorganisms and their metabolites, and these differential metabolites are significantly correlated with differential intestinal microorganisms, which may in turn affect the production performance of weaned piglets.

## 1. Introduction

Weaned piglets represent a crucial segment of the pig production industry, but they are particularly vulnerable to stress during the weaning process. This stress can adversely affect their growth performance, ultimately leading to reduced economic benefits for pig farms. Our previous studies have indicated that when weaned piglets are given a specific dose of chloroquine (CQ) to inhibit autophagy, this improves their production performance and relieves weaning pressure, while piglets fed a specific dose of rapamycin (RAPA) in the diet exhibited activated autophagy and had a lower final body weight (BW) and average daily gain (ADG) than the control treatment [1]. RAPA is a specific inhibitor of mammalian rapamycin (mTOR) signaling pathway targets, which improves autophagy by inducing microtubule-associated protein light chain 3 (LC3) flux in vivo and in vitro [2]. CQ, a lysosomal inhibitor, has been shown to reverse autophagy by accumulating in lysosomes and interfering with the vacuolar H+ATPase [3]. Moreover, the inhibition of autophagy has been shown to alleviate the host’s inflammatory response and augment the activity of antioxidant enzymes in serum, thereby resolving the occurrence of oxidative stress in the host, in which gut function plays a well-established role [1].

Autophagy serves a protective role in cells by degrading and recycling proteins and organelles to maintain intracellular homeostasis. However, when autophagy’s mechanisms are disrupted or the autophagic flux is exceeded, this can lead to cell death [4]. The intestinal microbiota influences host autophagy through a variety of pathways and complex regulatory networks governing the autophagy mechanisms. In turn, autophagy and autophagy-related proteins can shape the gut microbiota [5]. There were increased levels of the autophagosome marker LC3-II in GF mice compared to SPF mice colonocytes, but the microbial metabolite butyrate inhibited the level of autophagy in the colons of GF mice [6]. Interestingly, the expression of some autophagy-related proteins (FoxO1, FoxO3, GABARAP and ATG7) and the LC3-II/LC3-I ratio were increased in newborn piglets receiving FMT [7]. Changes in the gut microbiota composition were observed in mice conditionally deficient for autophagy (Atg5^−/−^, Atg7^−/−^ and ATG16L1 T300A knock-in) in the gut [8,9,10]. Many human diseases are linked to an imbalance in microbial communities [11]. Early weaning reduced the growth performance, damaged jejunal morphology, disrupted immune profiles and oxidative balance and caused oxidative injury in piglets [12,13]. The intestinal microbes of piglets are in a critical period of construction when they are weaned [14]. Preliminary experimental results have demonstrated that improving the composition of the gut microbiome can improve anti-inflammatory and antioxidant capabilities, thereby alleviating weaning stress and increasing piglet production performance [15,16,17].

Therefore, exploring the relationship between intestinal autophagy and the gut microbiota in piglets during the weaning period is particularly valuable. Accordingly, in this study, we investigated the effects of the autophagy level on the gut microbiota and its metabolites in weaned piglet with the aim of analyzing the mechanism of autophagy affecting the production performance of weaned piglets from the microbiota perspective.

## 2. Methods and Materials

The RAPA and CQ components (purity ≥ 98%) were provided by Sangon Biotech Co., Ltd. (Shanghai, China).

### 2.1. Animal Treatment and Experimental Design

Eighteen 24-day-old Large White × Landrace weaned piglets (barrow, average initial body weight (BW) of 7.03 ± 0.57 kg) were divided into the following three treatments: CON (equal volume normal saline solution), RAPA (1 mg/kg BW) and CQ (10 mg/kg BW). There were 6 replicates per treatment and 1 pig per replicate. Piglets were given 7 mL of normal saline, RAPA or CQ orally once a day at 27 days of age. On the 7th day of the test (33 days of age), the total amount of solution was adjusted according to the average weight of each treatment, with the specific operation and feeding management informed by the preliminary experiment [1]. The basal diet was formulated according to the nutritional needs of weaned piglets (NRC, 2012) [18], as proposed in previous studies [19]. The trial period was 17 days; see Figure S1 for details.

### 2.2. Sample Collection

At the conclusion of the experiment, the piglets were humanely slaughtered by bloodletting following electrical stunning. Subsequently, we isolated the ileum (terminal ileum) and colon (middle colon), collected digesta samples with sterile centrifuge tubes and stored those immediately in liquid nitrogen at −80 °C for microbiome or metabolite analysis (Figure S2).

### 2.3. Analysis for Bacterial Microbiota by 16S rRNA

Using a Stool DNA Isolation Kit (Tiangen Biotech Co., Ltd., Beijing, China), we extracted the total genomic DNA from 18 ileal and 18 colonic digesta samples according to the manufacturer’s instructions [20]. A NanoDrop ND-1000 spectrophotometer (Thermo Fisher Scientific, Meridian Rd, Rockford, IL, USA) and agarose gel electrophoresis were used to determine the quantity and quality of extracted DNA, respectively. Amplification of 16S ribosomal RNA genes in bacterial V3~V4 regions was achieved using primer polymerase chain reaction (PCR) (341F 5′-barcode-CCTAYGGGRBGCASCAG-3′ and 806R 5′-GGACTACNNGGGTATCTAAT-3′). We mixed the same volume of 1X loading buffer (contained SYB green) with PCR products and operated electrophoresis on 2% agarose gel for detection [21]. PCR products were mixed in equidense ratios. Then, the mixed PCR products were purified with a Universal DNA Purification Kit (Tiangen, China, Catalog #: DP214). Sequencing libraries were generated using an NEB Next^®^ Ultra™ II FS DNA PCR-free Library Prep Kit (New England Biolabs, County Rd, Ipswich, MA, USA, Catalog #: E7430L) following the manufacturer’s recommendations, and indexes were added [22].

### 2.4. Metabolomic Analysis

#### 2.4.1. Sample Pre-Treatment Method

For each tissue sample (80 mg), we added 200 μL of ultra-pure water for homogenization, followed by the addition of 800 μL of methanol/acetonitrile (1:1, *v*/*v*). The mixture was vortexed for thorough mixing and then ultrasound fragmented at low temperatures. Protein precipitation was performed through incubation at −20 °C for 1 h. Subsequently, centrifugation was conducted at 13,000 rpm and 4 °C for 15 min. The supernatant was collected and lyophilized for storage at −80 °C until further use.

#### 2.4.2. Chromatographic Conditions

Samples were separated using an Agilent 1290 Infinity LC ultra-high-performance liquid chromatography system (UHPLC) with an HILIC (Hydrophilic Interaction Liquid Chromatography) column; the column temperature was set at 25 °C; the flow rate was 0.3 mL/min; and the injection volume was 2 μL. The mobile phase consisted of component A: water + 25 mM ammonium acetate + 25 mM ammonia, and component B: acetonitrile. The gradient elution program was as follows: 0–1 min, 95% B; 1–14 min, B linearly changed from 95% to 65%; 14–16 min, B linearly changed from 65% to 40%; 16–18 min, B maintained at 40%; 18–18.1 min, B linearly changed from 40% to 95%; and 18.1–23 min, B maintained at 95%. Throughout the analysis process, samples were stored in an autosampler at 4 °C [23]. To avoid an impact due to instrument detection signal fluctuations, continual sample analysis was conducted in a random order. Quality control samples were inserted into the sample sequence to monitor and evaluate the stability of the system and reliability of the experimental data.

#### 2.4.3. Q-TOF Mass Spectrometry Conditions

Both electrospray ionization (ESI) positive ion and negative ion modes were used for detection. After separation by UHPLC, mass spectrometry analysis was performed on the Triple TOF 5600 mass spectrometer (AB SCIEX).

The ESI source conditions following HILIC chromatographic separation were as follows: Ion Source Gas1 (Gas1): 60, Ion Source Gas2 (Gas2): 60, Curtain Gas (CUR): 30, source temperature: 600 °C, IonSapary Voltage Floating (ISVF) ± 5500 V (both modes), TOF MS scan *m*/*z* range: 60–1000 Da, product ion scan *m*/*z* range: 25–1000 Da, TOF MS scan accumulation time: 0.20 s/spectra, and product ion scan accumulation time: 0.05 s/spectra. Secondary mass spectrometry utilized information-dependent acquisition (IDA) and was acquired in the high-sensitivity mode. Declustering potential (DP): ± 60 V (both modes), and collision energy: 35 ± 15 eV. IDA settings: exclude isotopes within 4 Da, and candidate ions to monitor per cycle: 6.

### 2.5. Statistical Analysis

#### 2.5.1. Microbiota Data

The library was checked with Qubit and real-time PCR for quantification and a bioanalyzer for size distribution detection. Quantified libraries were pooled and sequenced on Illumina platforms, according to the effective library concentration and data amount required. Paired-end reads were assigned to samples based on their unique barcode and truncated by cutting off the barcode and primer sequence. Paired-end reads were merged using FLASH (V1.2.11, http://ccb.jhu.edu/software/FLASH/, accessed on 29 June 2018) [24], a very fast and accurate analysis tool, which was designed to merge paired-end reads when at least some of the reads overlapped the reads generated from the opposite end of the same DNA fragment, and the splicing sequences were called raw tags. Quality filtering on the raw tags was performed using the fastp (Version 0.23.1) software to obtain high-quality clean tags [25]. The tags were compared with the reference database (https://www.arb-silva.de/, accessed on 1 July 2024) using the UCHIME algorithm (http://www.drive5.com/usearch/manual/uchime_algo.html, accessed on 1 July 2024) to detect chimera sequences, and then the chimera sequences were removed [26]. Then the effective tags were finally obtained. Species annotation was performed using Uparse software (Uparse v7.0.1001, http://drive5.com/uparse/, accessed on 1 July 2024). In order to analyze the diversity, richness and uniformity of the communities in the sample, the alpha diversity was calculated from 4 indices in QIIME2: Observed_species, Shannon, Simpson and Chao1. Principal co-ordinate analysis (PCoA) was conducted with the ade4 package and the ggplot2 package in R software (Version 4.0.3). ANOSIM and MRPP analysis are non-parametric tests that analyze the differences between high-dimensional data groups. They can test whether the differences between groups are significantly greater than the differences within a group, which can determine whether the grouping is meaningful. All of these were performed with the vegan package and the ggplot2 package within R. LEfSe is widely used to discover biomarkers, and it can reveal metagenomic characteristics. To achieve this, an exclusive package named lefse was utilized.

#### 2.5.2. Metabolomic Data

Raw data were converted into the mzXML format using ProteoWizard and then subjected to peak alignment, retention time correction and peak area extraction using the XCMS program. Metabolite structure identification was achieved through accurate mass number matching (<25 ppm) and secondary spectrometric matching, with a search against an in-house database. For the data extracted by XCMS, ion peaks that accounted for more than two-thirds of group totals were removed. Pattern recognition was performed using SIMCA-P 14.1 software (Umetrics, Umea, Sweden). Following Pareto scaling pre-treatment of the data, multivariate statistical analysis was conducted including unsupervised principal component analysis (PCA) and supervised partial least squares discriminant analysis (PLS-DA). Univariate statistical analyses included Student’s t-test and fold-change analysis. Heat maps were drawn using R software. Differential compounds were defined as *p*  <  0.05 and VIP  >  1. Metabolic pathway enrichment analysis of differential metabolites was performed based on the KEGG database. Metabolic pathways with *p*  <  0.05 were significantly enriched by differential metabolites.

The data of partial differential metabolites were analyzed by the independent-samples t-test procedure of SPSS 26.0 (SPSS Inc., Chicago, IL, USA), and each piglet was considered a statistical unit. The data are presented as mean values with the standard error of the total mean (SEM). In this paper, *p* > 0.05 means the difference is not significant, *p* < 0.05 means the difference is significant and 0.05 < *p* < 0.10 means there is a difference trend.

## 3. Results

### 3.1. Gut Microbiota Diversity

**Figure 1:**
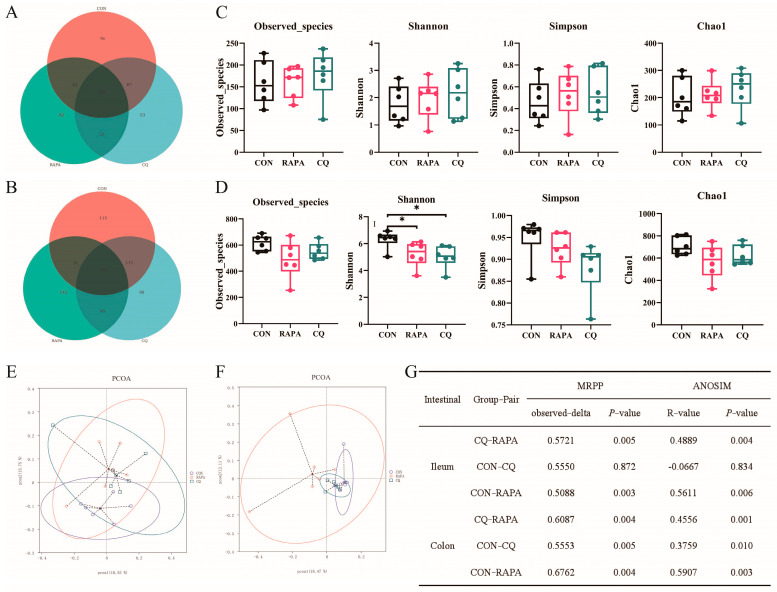
The *α*− and *β*−diversity indexes of the ileum and colon from 16s rRNA gene sequence analysis. Effects of different levels of autophagy on the ileac (**A**) and cecal (**B**) microbes in operational taxonomic units (OTUs) in piglets. α−diversity indexes including the Observed_species, Shannon, Simpson and Chao1 indexes of ileac (**C**) and colonic (**D**) microbes in piglets were observed. The PCoA results of ileac and colonic microbes in piglets are shown in (**E**,**F**), and the results of the MRPP and ANOSIM analysis are presented in (**G**). An individual piglet was regarded as the experimental unit (*n* = 6). CON, saline; RAPA, rapamycin; CQ, chloroquine. * indicates *p* < 0.05.

In the ileum, we identified 76, 82 and 53 unique OTUs in the CON, RAPA and CQ groups, respectively (Figure 1A). In the colon, there were 115, 142 and 66 unique OTUs in the CON, RAPA and CQ groups, respectively (Figure 1B). In the ileum contents, there were no significant differences in α-diversity indicators among any of the groups (*p* > 0.05) (Figure 1C). But the Shannon index of the colon in the CON group was significantly higher than that in the RAPA and CQ groups (*p* < 0.05) (Figure 1D). The β-diversity indexes of the ileum and colon contents, containing PCoA, MRPP and ANOSIM, showed that there were significant differences in microbial composition among the groups (*p* < 0.05) (Figure 1E–G).

### 3.2. Differential Gut Microbes

**Figure 2:**
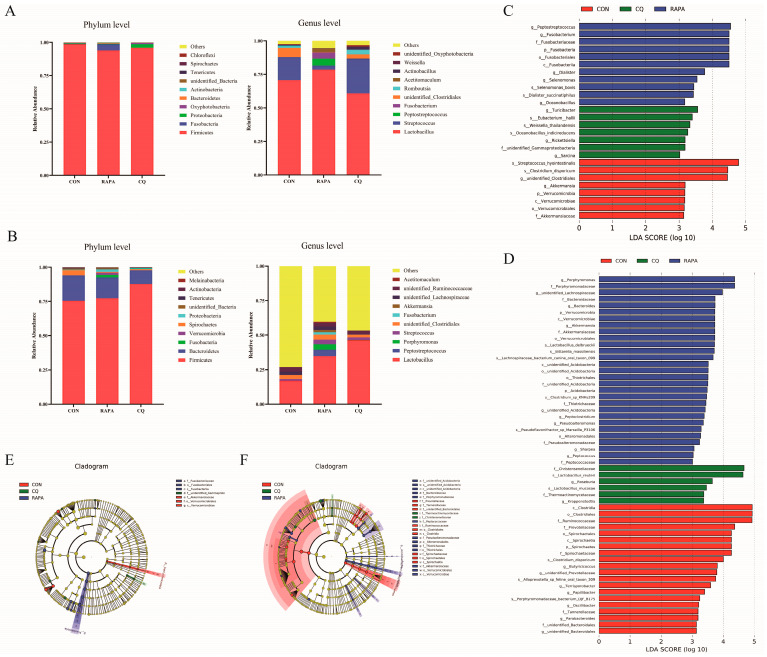
The relative species abundances of the ileum (**A**) and the colon (**B**) contents at the phylum level and the genus level. LEfSe analysis showed the significantly changed bacteria among the study groups in the ileum (**C**,**E**) and the colon (**D**,**F**). An individual piglet was regarded as the experimental unit (*n* = 6). CON, saline; RAPA, rapamycin; CQ, chloroquine.

The results indicated that the Firmicutes dominated in the ileum, while both the Firmicutes and Bacteroidetes dominated in the colon at the phylum level, accounting for more than 90% of the gut microbes. At the genus level, *Lactobacillus* accounted for the main microbes found both in the ileum and the colon (Figure 2A,B).

At the phylum level, the proportions of Fusobacteria in the ileum and Verrucomicrobia and Acidobacteria in the colon were enhanced in the RAPA group hen compared with the other groups (Figure 2C–F). At the gene level, the relative levels of abundance of *Peptostreptococcus*, *Fusobacterium*, *Dialister*, *Selenomonas* and *Oceanobacillus* in the ileum and *Porphyromonas*, *Bacteroides*, *unidentified_Lachnospiraceae*, *Akkermansia*, *Sharpea*, *Peptococcus*, *Pseudoalteromonas*, *Peptoclostridium* and *unidentified_Acidobacteria* in the colon were improved in piglets fed the RAPA diet, whereas the relative levels of abundance of *Turicibacter*, *Rickettsiella* and *Sarcina* in the ileum and *Roseburia* and *Kroppenstedtia* in the colon were increased in the CQ group (Figure 2C–F).

### 3.3. Ileac Differential Metabolites

**Figure 3:**
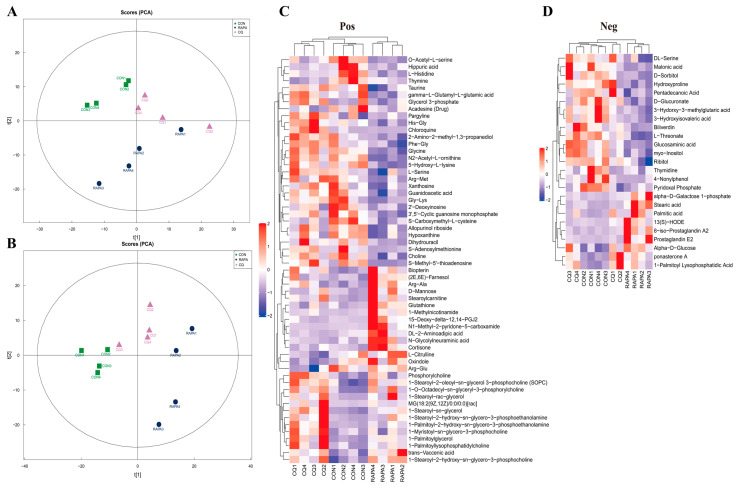
PCA scores measured by multivariate statistical analysis in POS and NEG models (**A**,**B**). Ileal differential metabolites in POS and NEG models (**C**,**D**). An individual piglet was regarded as the experimental unit (*n* = 4). CON, saline; RAPA, rapamycin; CQ, chloroquine.

The QC samples were closely distributed and highly correlated, indicating that the whole detection process is stable (Figure S3A–F). The PCA scores measured by multivariate statistical analysis in both positive (POS) and negative (NEG) models showed that the samples of ileal contents among groups could be easily divided into clusters (Figure 3A,B). We identified 58 positive metabolites and 25 negative metabolites that differed among the groups, mainly amino acid and lipid-associated metabolites (Figure 3C,D).

### 3.4. Ileac Differential Metabolites’ KEGG Enrichment

**Figure 4:**
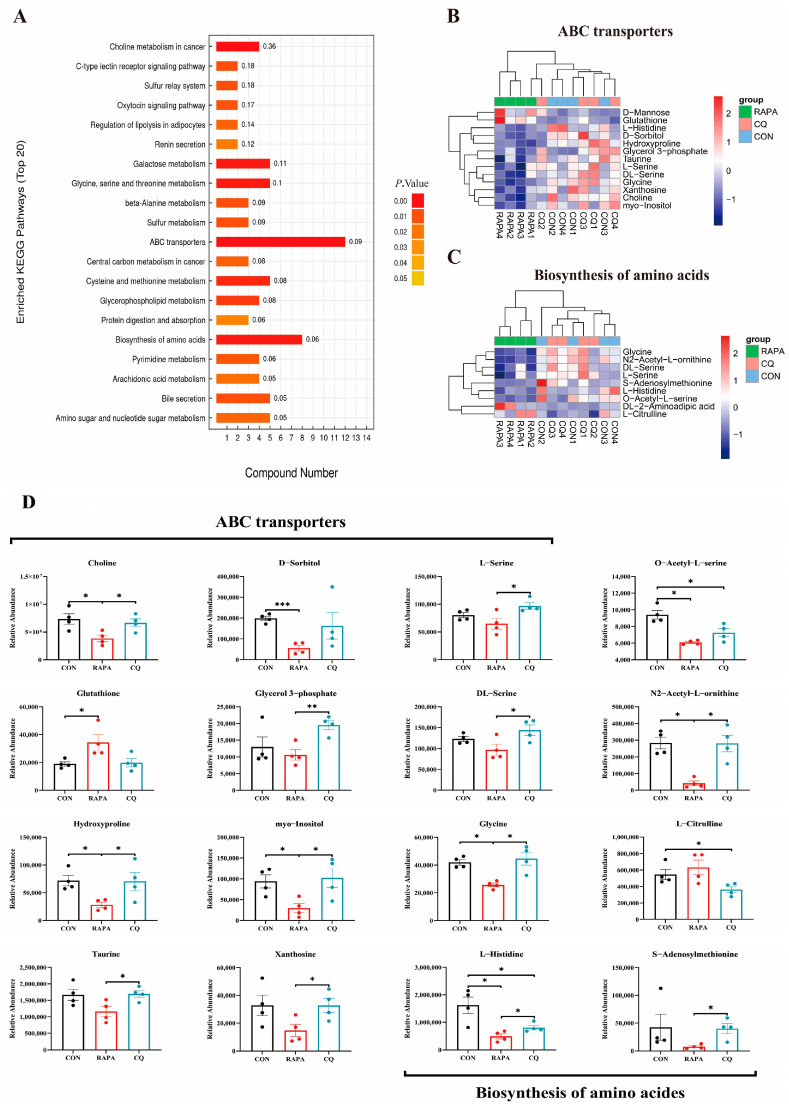
The top 20 KEGG pathways of differential metabolites in ileal contents were enriched (**A**). Heatmaps (**B**,**C**) and T-test results (**D**) of differential metabolites in the ABC transporter pathway and biosynthesis of amino acids pathway. An individual piglet was regarded as the experimental unit (*n* = 4). CON, saline; RAPA, rapamycin; CQ, chloroquine. * indicates *p* < 0.05; ** indicates 0.01 < *p* < 0.05; *** indicates *p* < 0.01.

In the KEGG pathway enrichment analysis based on differential metabolites, we observed an enrichment of two pathways (ABC transporters and biosynthesis of amino acids), which mainly consist of amino acid metabolites (Figure 4A–C). When compared with the CON group, the relative levels of abundance of choline, D-sorbitol, O-acetyl-L-serine, N2-acetyl-L-ornithine, hydroxyproline, myo-Inositol, glycine and L-histidine were decreased and the relative abundance of glutathione was increased in the RAPA group (*p* < 0.05) (Figure 4D). Additionally, the relative levels of abundance of O-acetyl-L-serine, L-citrulline and L-histidine were reduced in the CQ group compared to the CON group (*p* < 0.05) (Figure 4D). Meanwhile, the relative levels of abundance of choline, L-serine, glycerol 3-phosphate, DL-serine, N2-acetyl-L-ornithine, hydroxyproline, myo-Inositol, glycine, taurine, xanthosine, L-histidine and S-adenosylmethionine in the RAPA group were significantly lower than those in the CQ group (*p* < 0.05) (Figure 4D).

### 3.5. Correlation Analysis of Gut Differential Microbes and Metabolites

**Figure 5:**
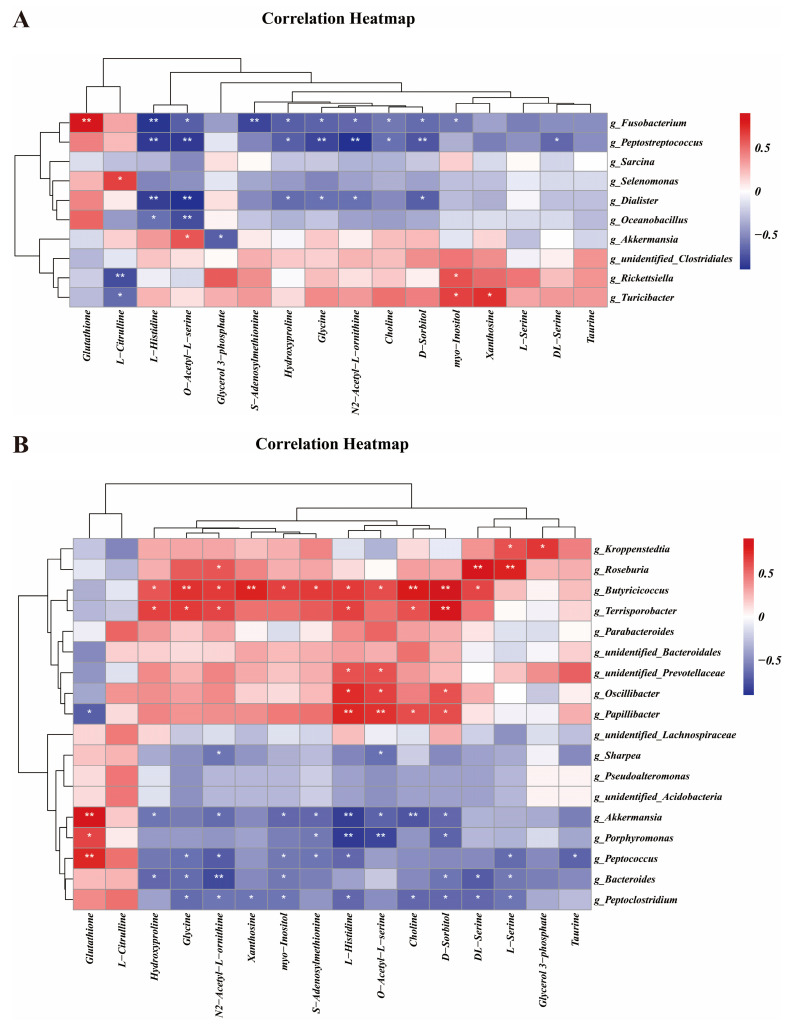
Correlation analysis between different microorganisms of the ileum (**A**) and the colon (**B**) and different metabolites of the ileum. An individual piglet was regarded as the experimental unit (*n* = 4). CON, saline; RAPA, rapamycin; CQ, chloroquine. * indicates *p* < 0.05; ** indicates *p* < 0.01.

The results showed that ileal *Fusobacterium*, *Peptostreptococcus* and *Dialister* were significantly negatively correlated with ileal L-histidine, O-acetyl-L-serine, hydroxyproline, glycine, N2-acetyl-L-ornithine and D-sorbitol (*p* < 0.05) (Figure 5A). Furthermore, ileal *Fusobacterium* was significantly negatively correlated with S-adenosylmethionine, choline and myo-inositol, but significantly positively correlated with glutathione (*p* < 0.05). Ileal *Peptostreptococcus* was significantly negatively correlated with choline and DL-serine (*p* < 0.05). In addition, ileal *Selenomonas* was significantly positively correlated with L-citrulline (*p* < 0.05). Moreover, ileal *Oceanobacillus* was significantly negatively correlated with L-histidine and O-acetyl-L-serine (*p* < 0.05). Furthermore, ileal *Rickettsiella* and *Turicibacter* were significantly positively correlated with myo-Inositol and xanthosine, whereas they were significantly negatively correlated with L-citrulline (*p* < 0.05).

In the colon, the microorganisms with a higher abundance in the CQ group, including *Kroppenstedtia* and *Roseburia*, were significantly positively associated with ileal N2-Acetyl-L-ornithine, DL-serine, L-serine and glycerol 3-phosphate (*p* < 0.05) (Figure 5B). Interestingly, as in the ileum, colonic microbes with a higher abundance in the RAPA group, including *Sharpea*, *Akkermansia*, *Porphyromonas*, *Peptococcus*, *Bacteroides* and *Peptoclostridium*, were significantly negatively associated with ileal hydroxyproline, glycine, N2-acetyl-L-ornithine, xanthosine, myo-inositol, S-adenosylmethionine, L-histidine, O-acetyl-L-serine, choline, D-sorbitol, DL-serine, L-serine and taurine, but significantly positively associated with ileal glutathione (*p* < 0.05).

## 4. Discussion

**Figure 6:**
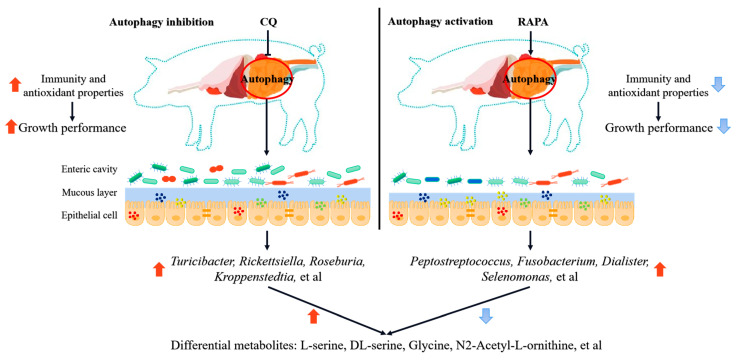
Autophagy activation or inhibition of regulation of intestinal microbes and their metabolites alleviating weaning stress in piglets.

The health, growth and feed intake of pigs were reduced once the intestinal and immune systems became dysfunctional during weaning. Inflammation and oxidative stress are common events in piglets under weaning stress [27,28]. When the body is stressed, it can relieve itself through autophagy, but excessive autophagy exacerbates the stress response and worsens the inflammatory response and oxidative stress state of the body [4]. Microorganisms in the gut not only play an indispensable role in the development of inflammation and oxidative stress but also in the regulation of autophagy [27,28,29,30]. In our study, the intestinal microbial composition of weaned piglets was affected by the level of intestinal autophagy and also affected the composition of intestinal metabolites. These changes may affect the production performance of piglets by influencing the immune and antioxidant capacity [1] (Figure 6).

In detail, regardless of whether treated with autophagy inhibitors or activators, the α-diversity of the colonic flora of piglets was reduced in this study. In addition, there were significant differences in microbial composition between the ileum and colon groups, which indicated that autophagy affects the composition of the gut microbiota [6]. Secondly, we found that at the genus level, *Lactobacillus* was the dominant bacterium in both the ileum and colon of the weaned piglets, and the relative abundance of *Lactobacillus* increased in the CQ group in the colon. *Lactobacillus*, as a beneficial bacterium, can significantly improve inflammation and oxidative stress damage in the body [31,32,33]. Furthermore, the levels of abundance of *Turicibacter* in the ileum and *Roseburia* in the colon were significantly higher in piglets treated with CQ than in the other groups. As is commonly known, *Turicibacter* alleviates inflammation and oxidative stress damage by regulating bile acid metabolism [34,35], while *Roseburia* regulates this damage through short-chain fatty acids [36]. Therefore, the change in the microbial composition could be one of the most important factors in inhibiting intestinal autophagy in weanling piglets treated with CQ to improve host inflammation and the oxidative stress state, thereby increasing piglet production performance. In addition, RAPA activation of the intestinal autophagy of weaned piglets also changed the composition of gut microbiota, such as increasing the relative levels of abundance of *Peptostreptococcus* and *Fusobacterium*, which were found to increase significantly in colon cancer patients [37,38]. The increases in these bacteria may also be one of the reasons for the inflammation and oxidative stress of weaned piglets after RAPA treatment, thus reducing the production performance of piglets. Moreover, changes in intestinal microorganisms can also cause the destruction of the intestinal barrier, such as through alteration of the intestinal morphology or downregulation of intestinal-barrier-related protein expression (ZO-1, occludin, integrin, E-cadherin). These changes may also be one of the factors that regulate the body’s oxidative stress and inflammatory response through the level of autophagy [1].

The level of intestinal autophagy in weaned piglets changed the composition of intestinal microbes, and we found that their metabolites also changed significantly. Significant differences were found among the CON, CQ and RAPA groups in the metabolites of the ileum contents. According to KEGG enrichment analysis, differential metabolites were mainly concentrated in metabolic pathways such as ABC transporters and biosynthesis of amino acids. We found that metabolites such as L-serine, DL-serine, glycine, taurine and N2-acetyl-L-ornithine were significantly higher in the CQ group than in the RAPA group, and these metabolites all play an important role in regulating inflammation and oxidative stress in the body [39,40,41,42]. Serine can affect the elimination of ROS by participating in the synthesis of GSH, thus improving the oxidative stress of the body [39,40]. Wang et al. found that glycine can inhibit oxidative-stress-induced apoptosis of IPEC-1 [41]. Arginine can protect cells from apoptosis triggered by LPS-induced oxidative damage, and ornithine, as a reaction product of arginine, can reduce arginine levels under certain circumstances [42]. Changes in gut microbes drive changes in their metabolites, and these changes may be closely related to the effects of autophagy on piglet growth performance by regulating the inflammatory response and oxidative stress. As we know, butyrate, a metabolite of the gut microbiota, can improve the gut health and increase the antioxidant capacity of piglets, thereby improving growth performance [43,44].

To further investigate the relationship between gut microbes and gut metabolites, we conducted a correlation analysis of various microbes and metabolites. Differential microorganisms, which had high abundance values in the RAPA group, included *Peptostreptococcus*, *Fusobacterium* and *Dialister* in the ileum, as well as *Porphyromonas* and *Bacteroides* in the colon. These microorganisms were found to be significantly negatively correlated with the metabolites other than glutathione and L-citrulline. However, the ileac *Turicibacter* and *Rickettsiella* were significantly negatively correlated with L-citrulline, while they were positively correlated with myo-inositol and xanthosine in the CQ group; meanwhile, the colonic *Roseburia* and *Kroppenstedtia* were significantly positively correlated with L-serine, DL-serine and N2-acetyl-L-ornithine. These metabolites are closely associated with oxidative stress and inflammation in the body [39,40,41,42]. The regulatory relationship between intestinal microorganisms and metabolites may be directly regulated by the decomposition of upstream nutrients by microorganisms or indirectly influenced by metabolites after microbial decomposition to regulate other metabolites, such as indole-3-propenoic acid (IDA). The metabolite of tryptophan decomposition by the intestinal microorganism *P. anaerobius* promotes colorectal cancer [45]. Moreover, symbiotic bacteria *Roseburia intestinalis* ameliorates M-MFT-induced mastitis and microbial dysregulation in the gut and mammary glands by producing butyrate, which is associated with inflammation signal suppression and barrier repair, and limits bacterial translocation [46]. This offers further evidence that autophagy can regulate inflammation and oxidative stress by influencing the composition of the gut microbiota and its metabolites, thus affecting the piglets’ production performance [32,33,34,35,39,40,41,42].

## 5. Conclusions

According to our study, the inhibition of autophagy by CQ can regulate the gut microbial composition, such as increasing the relative abundance of *Turicibacter*, *Rickettsiella* and *Sarcina* in the ileum and *Roseburia* and *Kroppenstedtia* in the colon. Meanwhile, the functional enrichment of differential metabolites is mainly concentrated on the ABC transporters pathway and biosynthesis of amino acids pathway, and these differential metabolites are significantly correlated with differential intestinal microorganisms, suggesting that autophagy inhibition may not only change the composition of gut microbiota but also regulate gut microbiota metabolism, thereby alleviating weaning stress and improving piglet production performance. However, at present, the application of autophagy regulation in pigs is subject to many limitations. First of all, the research on the autophagy mechanism of pigs has been methodologically imperfect. The second issue is the high cost of autophagy inhibitor or activator products. Against that background, the analysis of intestinal microbes and their metabolites in this study provides a new idea for future research on the regulatory role of autophagy in piglets. However, there were still certain methodological problems in the current study, and follow-up research will be conducted, with more in-depth investigations of how autophagy regulates key microorganisms or metabolites in the intestines of weaned piglets.

## Data Availability

The 16S rRNA sequencing raw data from this study were deposited in the NCBI (https://www.ncbi.nlm.nih.gov, accessed on 29 April 2024). The BioProject accession numbers for the 16S rRNA sequencing are PRJNA1100210 and PRJNA1105661.

## Supplementary Materials

The following supporting information can be downloaded at https://www.mdpi.com/article/10.3390/vetsci11080333/s1: Figure S1. Experimental design of weaned piglets. An individual piglet was regarded as the experimental unit (*n* = 6). CON, saline; RAPA, rapamycin; CQ, chloroquine. Figure S2. Schematic diagram of 16s rRNA analysis and metabolomics analysis. CON, saline; RAPA, rapamycin; CQ, chloroquine. Figure S3. PLS-DA model validation between groups in POS and NEG models. A and D represent the PLS-DA model validation results of the CON group and the RAPA group in the POS and NEG models, respectively. B and E represent the PLS-DA model validation results of the CON group and the CQ group in the POS and NEG models, respectively. C and F represent the PLS-DA model validation results of the RAPA group and the CQ group in the POS and NEG models, respectively. CON, saline; RAPA, rapamycin; CQ, chloroquine.
