# Supplementary material for: Chloroquine Downregulation of Intestinal Autophagy Changed Intestinal Microbial Community Compositions and Metabolite Profiles in Piglets
Source: Vet Sci. 2024 Jul 25;11(8):333. doi: 10.3390/vetsci11080333 (PMC11360670; doi:10.3390/vetsci11080333)
Supplement: Supplementary file 1 [file vetsci-11-00333-s001.zip › vetsci-3066959-supplementary.pdf]

Supplemental Figures

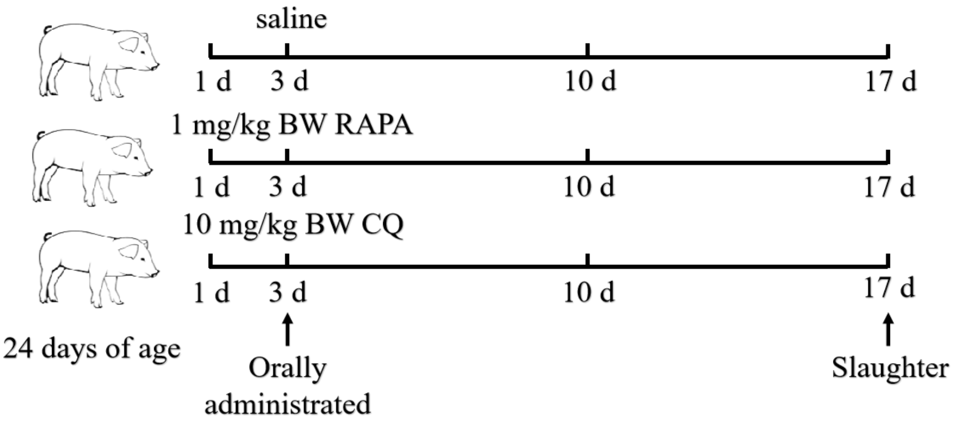

Figure S1. Experimental design of weaned piglets. The individual piglet was regarded as the experimental unit (n = 6). CON, administer saline; RAPA, take rapamycin; CQ, take chloroquine.

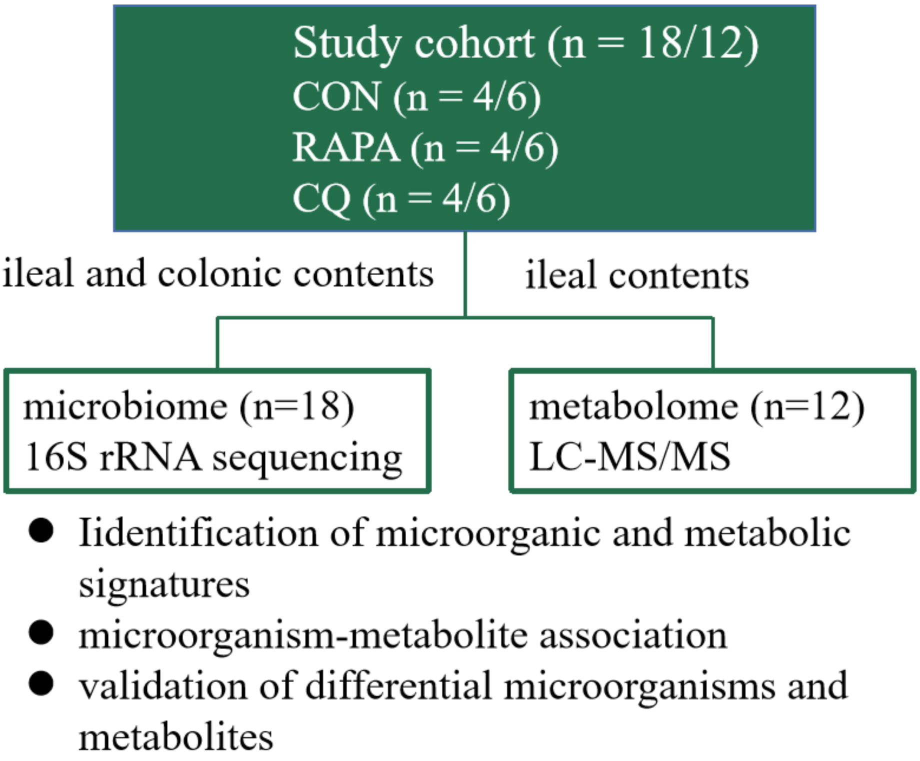

Figure S2. Schematic diagram of 16s rRNA analysis and metabolomics analysis. CON, administer saline; RAPA, take rapamycin; CQ, take chloroquine.

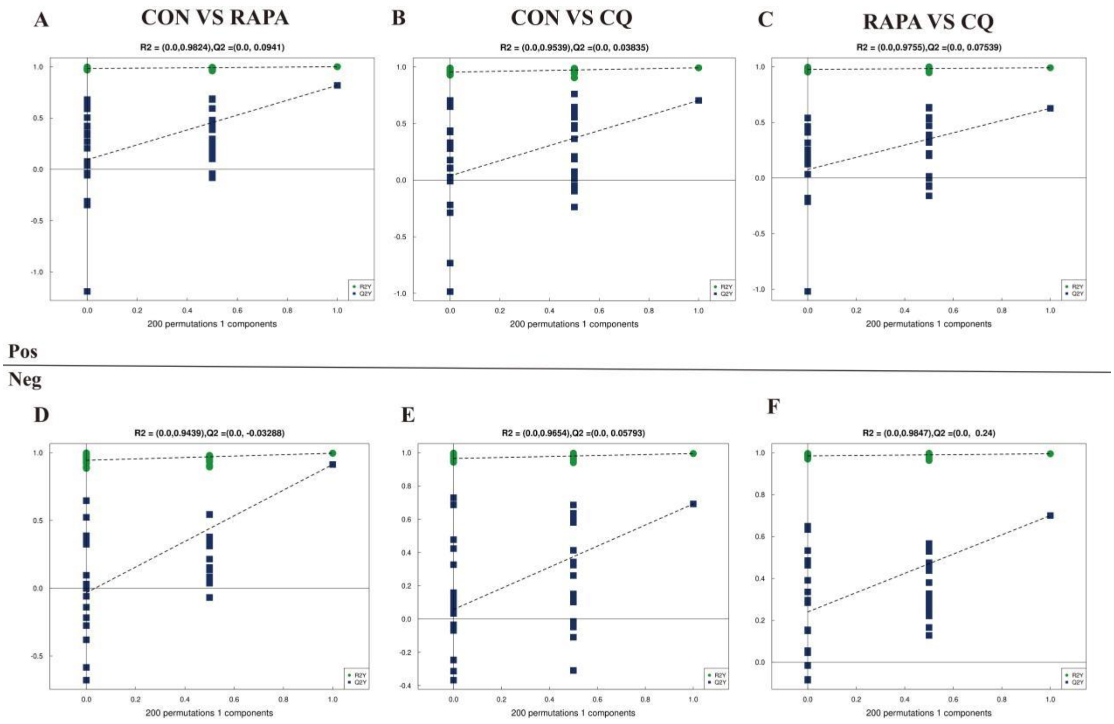

Figure S3. The PLS-DA model validation between groups in POS and NEG models. A and D represent the PLS-DA model validation results of CON group and RAPA group at POS and NEG, respectively. B and E represent the PLS-DA model validation results of CON group and CQ group at POS and NEG, respectively. C and F represent the PLS-DA model validation results of RAPA group and CQ group at POS and NEG, respectively. CON, administer saline; RAPA, take rapamycin; CQ, take chloroquine.
